# Comorbidities of anxiety disorders in bipolar patients: therapeutic complexity

**DOI:** 10.1192/j.eurpsy.2023.973

**Published:** 2023-07-19

**Authors:** S. Bahetta, N. El moussaoui

**Affiliations:** Psychiatric hospital arrazi, Sale; 2 Psychiatric hospital arrazi, Morocco, Morocco

## Abstract

**Introduction:**

Numerous clinical and epidemiological studies show that the rate of comorbidity of anxiety disorders is high in bipolar patients compared to the general population. This is associated with a poorer prognosis, poorer functioning and higher suicidal risk. Anxiety comorbidity should therefore be carefully investigated.

**Objectives:**

Our main objectives are to explore the therapeutic complexity of anxiety disorders in patients with bipolar disorder To investigate the existence of psycho-pathological links and vulnerabilities between bipolar disorder and anxiety disorders.

**Methods:**

through a clinical vignette and a review of the existing literature on the comorbidity of anxiety disorders and bipolar disorders, and the resulting therapeutic issues

**Results:**

Anxiety comorbidity is quite common in the bipolar population. In the American National Comorbidity Survey (NCS), lifetime comorbidity is close to 90%. Two recent French clinical studies show the existence of at least one anxiety disorder in approximately 25% of bipolar subjects (24% and 27.2%), which will have an impact on the course of the bipolar disorder, with a particular increase in the risk of suicide, hence the importance of adequate treatment. This treatment faces two obstacles: the risk of manic episodes under antidepressants and the risk of dependence on benzodiazepines. Emphasis is also placed on non-drug approaches, including cognitive-behavioural and psycho-educational therapies.

**Conclusions:**

Anxiety comorbidity is not without consequence on the evolution of bipolar disorder. Its particularly high prevalence means that it cannot be neglected or ignored in current practice.

**Disclosure of Interest:**

None Declared

